# 101 Machine Learning Algorithms for Mining Esophageal Squamous Cell Carcinoma Neoantigen Prognostic Models in Single-Cell Data

**DOI:** 10.3390/ijms26073373

**Published:** 2025-04-04

**Authors:** Yingjie Sun, Yuheng Tang, Qi Qi, Jianyu Pang, Yongzhi Chen, Hui Wang, Jiaxiang Liang, Wenru Tang

**Affiliations:** Laboratory of Molecular Genetics of Aging & Tumor, Medicine School, Kunming University of Science and Technology, No. 727, Jingming South Road, Kunming 650500, China; sunyj6039@163.com (Y.S.); t1023063288@163.com (Y.T.); qiqi12047@163.com (Q.Q.); jianyu_0898@163.com (J.P.); cyz1206414925@163.com (Y.C.); huiwang266@163.com (H.W.); liangjiaxiang2001@163.com (J.L.)

**Keywords:** ESCC, single-cell, machine learning, neoantigen-related genes, molecular docking of drugs

## Abstract

Esophageal squamous cell carcinoma (ESCC) is one of the most aggressive malignant tumors in the digestive tract, characterized by a high recurrence rate and inadequate immunotherapy options. We analyzed mutation data of ESCC from public databases and employed 10 machine learning algorithms to generate 101 algorithm combinations. Based on the optimal range determined by the concordance index, we randomly selected one combination from the best-performing algorithms to construct a prognostic model consisting of five genes (*DLX5*, *MAGEA4*, *PMEPA1*, *RCN1*, and *TIMP1*). By validating the correlation between the prognostic model and antigen-presenting cells (APCs), we revealed the antigen-presentation efficacy of the model. Through the analysis of immune infiltration in ESCC, we uncovered the mechanisms of immune evasion associated with the disease. In addition, we examined the potential impact of the five prognostic genes on ESCC progression. Based on these insights, we identified anti-tumor small-molecule compounds targeting these prognostic genes. This study primarily simulates the tumor microenvironment (TME) and antigen presentation processes in ESCC patients, predicting the role of the neoantigen-based prognostic model in ESCC patients and their potential responses to immunotherapy. These results suggest a potential approach for identifying therapeutic targets in ESCC, which may contribute to the development of more effective treatment strategies.

## 1. Introduction

Esophageal cancer (ESCA) is one of the most aggressive malignant tumors of the human digestive system. It is the sixth leading cause of cancer-related deaths and the eighth most common cancer worldwide, with a five-year survival rate ranging from 15% to 25% [1]. ESCA is classified into two subtypes: ESCC and esophageal adenocarcinoma (EAC) [2]. ESCC is primarily prevalent in East Asia, South Asia, and Sub-Saharan Africa, while EAC is more commonly found in Northern Europe and North America [3]. ESCC is primarily caused by smoking, alcohol consumption, and the intake of excessively hot foods. The development of this disease shows a regional genetic predisposition in developing countries [4]. The highest incidence of ESCC is in East Asia, where it accounts for more than 90% of ESCA [5]. Early-stage ESCC can be treated through endoscopic resection. However, for advanced-stage patients, surgery alone is not sufficient, and postoperative chemotherapy remains the best current adjunctive treatment [6]. ESCC in the field of immunotherapy is currently limited to immune checkpoint inhibitor therapies. Developing more diverse immunotherapy approaches is crucial for improving treatment outcomes [7].

In the past decade, cancer treatment has advanced rapidly. Following surgery, chemotherapy, and radiotherapy, immunotherapy has emerged as a new approach to cancer treatment [8]. Immunotherapy has progressed from the initial cytokine interference techniques to immune checkpoint blockade therapy and adoptive cell therapy and now to the current development of cancer vaccines [9]. Cancer vaccines aim to amplify tumor-specific T-cell responses by actively introducing attenuated antigens into the body. The exogenous tumor antigens in cancer vaccines can be delivered to APCs, particularly dendritic cells (DCs), to induce CD4+ T-cell and cytotoxic T lymphocyte (CTL) responses, thereby eliminating tumor cells [10]. Neoantigens in TME are undoubtedly ideal candidates for the development of tumor vaccines. These neoantigens, generated by somatic mutations in the cancer cell genome, typically exhibit high immunogenicity and can serve as tumor-specific immune targets [11]. Since neoantigens in patients with the same type of cancer are highly specific, a flexible and efficient vaccine technology is required to develop personalized tumor vaccines [12]. In the past two years, the rapid development of mRNA vaccine preparation technology during the COVID-19 pandemic has also laid the foundation for the maturation of tumor mRNA vaccine technology [13]. Current mRNA vaccines offer advantages such as low production costs and rapid manufacturing. Additionally, compared with DNA vaccines, mRNA vaccines demonstrate good tolerability, typically manageable and short-lived adverse events, and the absence of genomic integration risks [14,15,16]. mRNA vaccines can be flexibly customized to provide personalized treatment for different patients and can be combined with other therapeutic approaches to treat ESCC effectively [17].

In this study, we are focused on discovering a neoantigen-based prognostic gene model for ESCC to improve the prognosis and treatment outcomes for patients. To overcome the limitations of small sample sizes, we first integrated two large scRNA-seq datasets. We then identified the copy number variation (CNV) epithelial cancer cell clusters in ESCC samples and used pseudotime analysis to explore immune responses and mutations in mutated CNV cancer cells at different stages. We intersected the mutation dataset with the upregulated genes in ESCC, and we applied 101 algorithms to screen for a prognostic model of ESCC neoantigens in patients. Finally, within the optimal model range, we chose the LASSO regression, which is commonly used in medical research, for modeling and improving model accuracy by integrating the elastic net and random seed methods. We examined the correlation between the prognostic model and APCs as well as MHC II molecules in the immune infiltration of ESCC to validate the antigen presentation mechanism of the prognostic model. Additionally, we explored the pathogenesis of ESCC by analyzing TME. Then, we employed univariate Cox and multivariate Cox analyses to correlate the model with various clinical characteristics of the patients, aiming to assess whether the model could serve as an independent prognostic factor for the patients. Finally, we conducted functional exploration and molecular docking studies on the pathogenic mechanisms of each gene in the prognostic model to investigate potential combined therapeutic approaches.

## 2. Results

### 2.1. Identification of Cell Types

**Figure 1:**
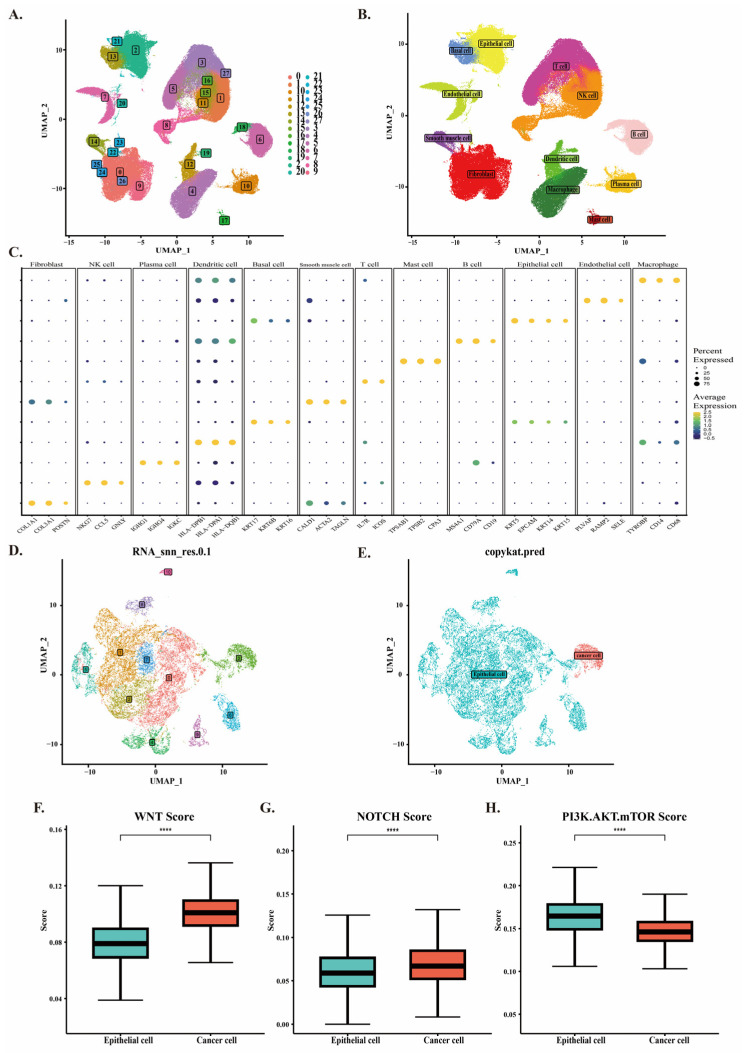
Single-cell Data Analysis and Cell Scoring Detection. (**A**) The 28 cell clusters obtained through dimensionality reduction and clustering. (**B**) Identification of 12 cell types through marker gene annotation. (**C**) Marker genes for each cell type. (**D**) The CopyKAT algorithm divides epithelial cells into 11 clusters. (**E**–**H**) The CopyKAT algorithm automatically identified clusters of cancer cells with aneuploidy mutations, and the WNT signaling pathway, NOTCH signaling pathway, and PI3K-AKT-mTOR signaling pathway scores between CNV cancer cells and malignant epithelial cells were analyzed. (**** *p* < 0.0001).

The technical workflow of this study (Figure S1A,B). First, we merged the datasets GSE145370 and GSE160269, resulting in a sample of 67 patients. After removing batch effects and performing quality control, 410,270 high-quality cells were selected. These merged data were then subjected to dimensionality reduction and clustering analysis, dividing the cells into 28 clusters. Through automated annotation and manual annotation analysis, we identified 12 cell subpopulations, including epithelial cells, basal cells, endothelial cells, fibroblasts, T cells, natural killer cells, B cells, plasma cells, mast cells, macrophages, and dendritic cells (Figure 1A,B). A bubble plot was used to display the specific genes in each cell cluster (Figure 1C).

Neoantigens are cancer-specific antigens that arise from somatic mutations in the genome of cancer cells. These mutations may exhibit high immunogenicity and are not subject to central tolerance mechanisms [11]. Through cell annotation, I identified a total of 48,164 epithelial cells. This is consistent with the number of malignant epithelial cells annotated by the original authors of these data [18]. Using the CopyKAT algorithm, I further extracted 3232 epithelial cancer cells with only CNV (Figure 1D,E and Figure S2). Subsequently, we performed pathway scoring for malignant epithelial cells and CNV cancer cells separately. The results showed that the scores of the WNT and NOTCH signaling pathways in CNV cancer cells were lower than those of the PI3K/AKT/mTOR signaling pathway (Figure S3A–C). However, compared with malignant epithelial cells, the WNT and NOTCH signaling pathways were significantly upregulated in CNV cancer cells (Figure 1F,G), indicating that CNV cancer cells are more likely to be influenced by these two pathways to promote enhanced proliferation, invasion, and metastasis. In contrast, the regulation of CNV cancer cells by the PI3K/AKT/mTOR signaling pathway appeared to have a relatively smaller impact compared with malignant epithelial cells (Figure 1H). We also examined the expression of ESCC cancer marker genes EPCAM and SFN in two cell populations (Figure S4A,B). The results revealed that EPCAM was highly expressed in CNV cancer cells, while SFN was expressed at low levels. Sensitivity testing was conducted by adjusting different thresholds of the CopyKAT algorithm, and the results demonstrated the stability of the CopyKAT algorithm (Figure S5). These results highlight the complexity of ESCC regulation, and through the CopyKAT algorithm, we screened out squamous epithelial cancer cells with copy number variation (CNV).

### 2.2. Mutation of CNV Cancer Cells at Different Developmental Stages

**Figure 2:**
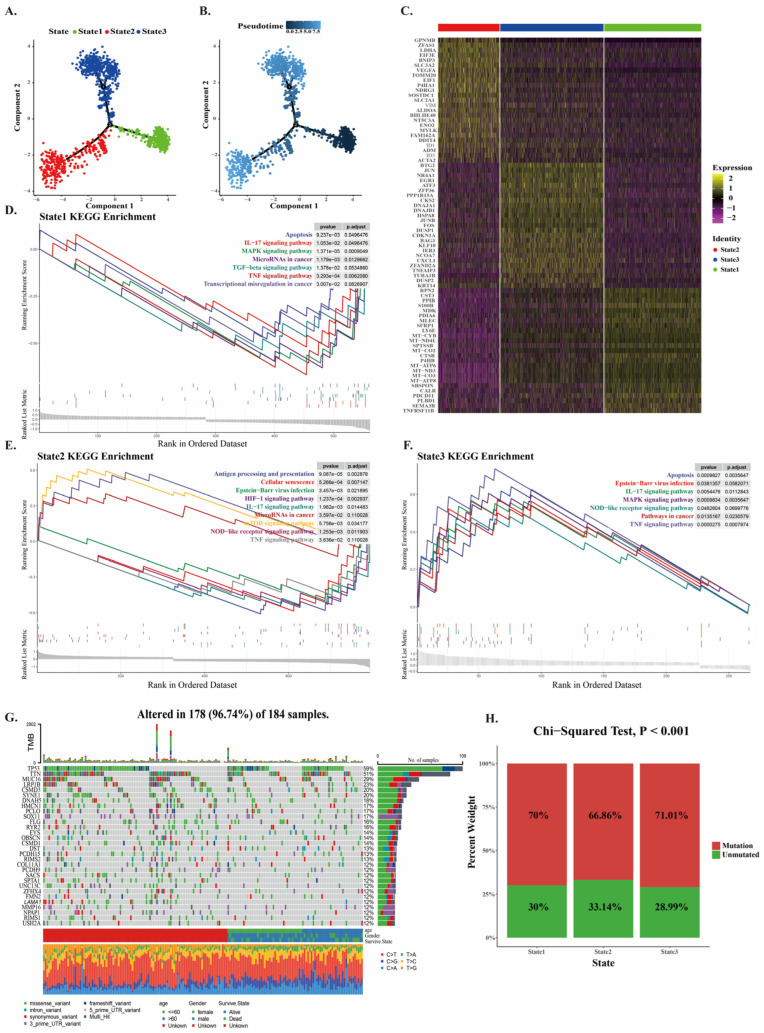
Pseudotime and mutation analysis of CNV cancer cells. (**A**,**B**) Based on pseudotime analysis, CNV cancer cells are divided into three distinct developmental states. (**C**) Heatmap displaying the top 30 mutated genes in each state. (**D**–**F**) GSVA analysis of pathway activity in the three developmental states of CNV cancer cells. (**G**) ESCC top 30 SNV genes and mutation types. (**H**) Mutation profiles of both SNV and CNV in the three developmental states of squamous epithelial cell carcinoma.

To explore the pathways and mutation of CNV cancer cells at different developmental stages, we performed pseudotime trajectory analysis on CNV cancer cells using the Monocle algorithm, demonstrating three distinct developmental states of CNV cancer cells during developmental stages (Figure 2A–C). Pathway enrichment analysis of the mutated gene sets obtained from pseudotime analysis of CNV cancer cells in the three developmental states revealed that cancer-related pathways such as the TNF signaling pathway and apoptosis pathway were downregulated in State 1, suggesting that State 1 may represent the initial stage of tumor development (Figure 2D). In State 2, the upregulation of pathways such as antigen presentation, mTOR signaling, and NOD-like receptor signaling suggests that cancer cells in this state are in an active growth and proliferation phase, with inflammatory responses present in the tumor. Tumor microenvironment-related antigens are recognized by antigen-presenting cells, triggering the antigen presentation mechanism (Figure 2E). In State 3, both cancer-related pathways and immune-related pathways are upregulated, indicating that CNV tumors at this stage are in an active state of growth and proliferation and may evade immune responses through certain mechanisms (Figure 2F). The analysis of SNVs in ESCC from TCGA revealed that mutations in TP53 lead to the loss of DNA repair and apoptosis capabilities in cells, thereby promoting the proliferation and development of cancer cells. Abnormalities in TNN significantly enhance the invasive and migratory abilities of cancer cells (Figure 2G). Additionally, further analysis showed that the SNV mutation rate in CNV cancer cells exceeds 65% across different developmental stages (Figure 2H). In summary, we investigated the growth environments and characteristic genes of CNV cancer cells at different developmental stages and identified characteristic mutation genes in ESCC cancer cells by intersected he feature genes with the SNV dataset from TCGA.

### 2.3. Construction of Neoantigen Prognostic Risk Model

**Figure 3:**
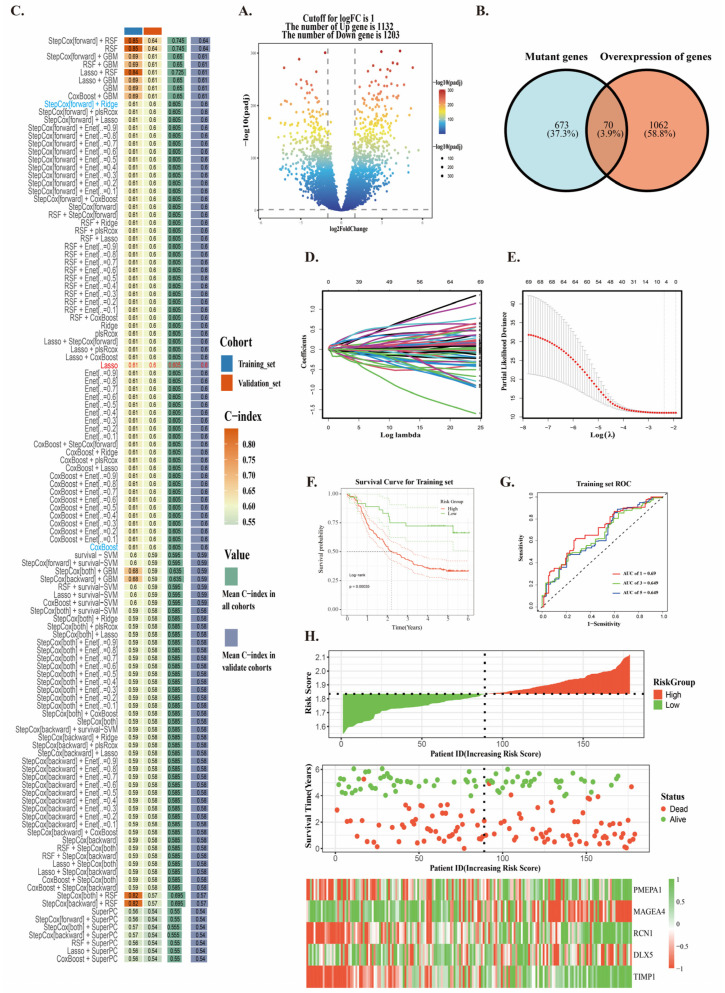
Construction and Validation of the Predictive Risk Model. (**A**) Differential analysis of ESCC. (**B**) Intersection of overexpressed genes and mutated genes. (**C**) Screening of prognostic risk models using 101 machine learning algorithms. (**D**,**E**) Construction of the prognostic risk model using single-line network optimization and LASSO. (**F**) KM survival analysis for the training set. (**G**) ROC curve for the training set. (**H**) Survival scatter plot for the training set.

We integrated bulk RNA-seq data of ESCC from the TCGA database and GEO database (Figure S6). To identify candidate genes suitable as targets for neoantigen mRNA vaccines, we plan to screen for significantly upregulated genes in cancer. These genes exhibit high expression levels in the tumor microenvironment and may encode immunogenic neoantigens. Therefore, we used the GSE53264 dataset and performed differential analysis on cancer samples and adjacent normal samples from patients. We filtered for upregulated genes with logFC > 1 and a *p*-value < 0.05, identifying a total of 1132 genes. These genes were defined as overexpressed genes. (Figure 3A). Subsequently, we intersected these overexpressed genes with the characteristic mutation gene set and ultimately selected 70 genes to construct the prognostic risk model (Figure 3B).

We used the GSE53624 dataset (*n* = 119) as the training set and merged the TCGA and GSE53622 datasets (*n* = 60) as the validation set. We also evaluated the model quality of StepCox [forward] + RSCF and the results did not meet expectations (Figure S7). Therefore, by analyzing the C-index of each machine learning combination in the training and validation sets, we identified the model combination with the smallest C-index difference to construct the prognostic model. From the results, we observed that the C-index values of the algorithm combinations between StepCox [forward] + Ridge and CoxBoost were not high. Consequently, we opted for LASSO regression, a method widely recognized in the medical field for its stability and computational efficiency, to build the neoantigen prognostic model. By integrating elastic net and random number methods, the neoantigen prognostic risk model achieved higher stability and superior performance (Figure 3B,C,E). Subsequently, we demonstrated that the neoantigen prognostic model achieved AUC values greater than 0.6 for 1-year, 3-year, and 5-year survival in both the training and validation sets (Figure 3G and Figure S8A–C), indicating that the neoantigen prognostic model has good predictive ability for survival risk in ESCC. The formula for the neoantigen prognostic risk model is as follows: Risk score = 0.0135 × PMEPA1 − 0.0245 × MAGEA4 + 0.0622 × RCN1 − 0.0071 × DLX5 + 0.0792 × TIMP1.

Based on the median neoantigen risk score, patients were divided into high-risk and low-risk groups. The results showed that the overall survival (OS) of patients in the high-risk group was significantly lower than that in the low-risk group (Figure 3F and Figure S8A). Additionally, the number of surviving patients in the low-risk zone is significantly higher than that in the high-risk zone, while the number of deceased patients in the high-risk zone is notably greater than in the low-risk zone. From the graph, we observe that PMEPA1, RCN1, and TIMP1 are highly expressed in the low-risk group, while the expression of MAGEA4 and DLX5 begins to increase significantly with the rise in risk scores (Figure 3H and Figure S8C). These results indicate that the neoantigen prognostic risk model score can serve as a predictive factor for patient prognosis.

### 2.4. The Antigen Presentation Effect and Immune Microenvironment of the Prognostic Risk Model

**Figure 4:**
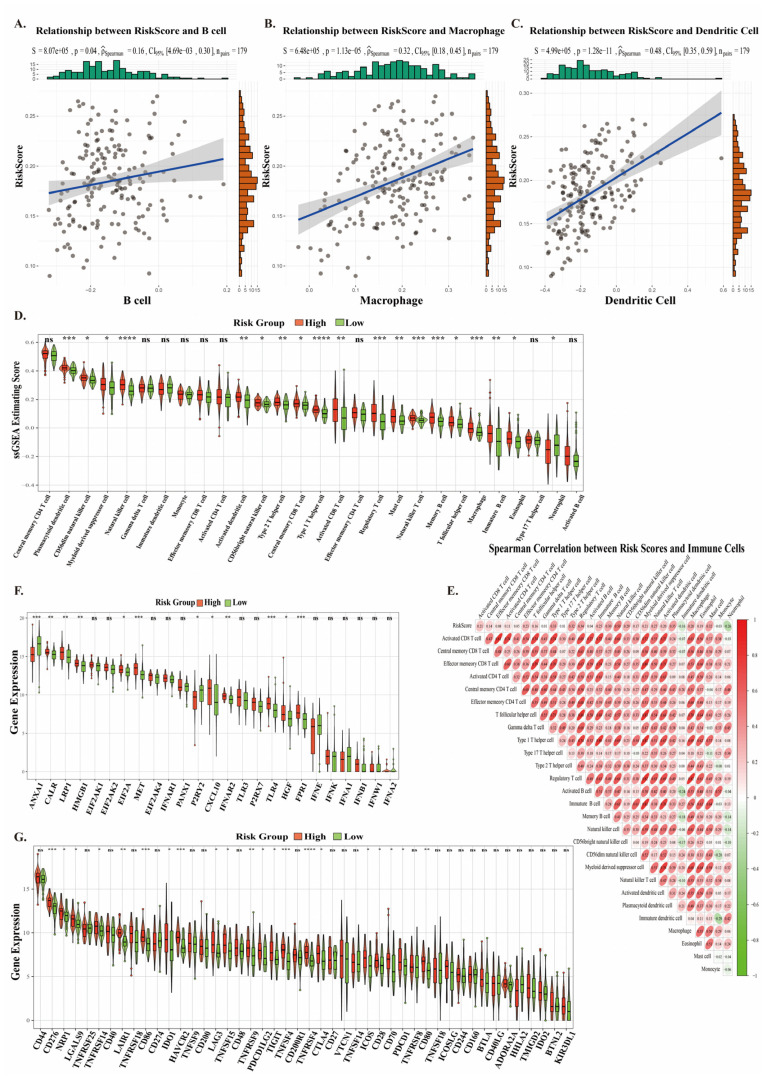
Immune Predictive Performance of the ESCC Prognostic Model. (**A**–**C**) Correlation between the ESCC prognostic model and immune infiltration of B cells, macrophages, and dendritic cells. (**D**) Abundance of 28 immune cell types in the high-risk and low-risk groups, stratified by the median risk score. (**E**) Spearman correlation analysis between the abundance of 28 immune cell types and the risk score. (**F**,**G**) Expression levels of ICI- and ICP-related genes in the cancer group and adjacent normal group. (* *p* < 0.05; ** *p* < 0.01; *** *p* < 0.001; **** *p* < 0.0001; NS stands for non-statistically significant).

To further investigate the relationship between the neoantigen prognostic model and antigen presentation, we found that the prognostic risk score was positively correlated with immune infiltration levels of dendritic cells, macrophages, and B cells (Figure 4A–C). Using ssGSEA, we analyzed the immune infiltration status of MHC class II-related genes in patients from the high-risk and low-risk groups of the neoantigen prognostic risk model. We found that patients in the high-risk group exhibited higher MHC class II immune infiltration scores (Figure S9A), suggesting that the antigen presentation effect is stronger in high-risk patients. As the risk score increases, the proportion of immune cells in patients also rises, indicating a more intense immune response (Figure 4E). Additionally, we analyzed the correlation between the five prognostic genes and APCs. We found that DLX5 showed a positive correlation with dendritic cells, while MAGEA4 exhibited no correlation with APCs. PMEPA1, RCN1, and TIMP1 were positively correlated with macrophages (Figure S9B–F). These findings suggest that the five prognostic genes are more likely to be recognized by dendritic cells and macrophages for antigen presentation, which also reflects the relatively lower correlation between the neoantigen prognostic model and B cell immune infiltration (Figure 4A). These results demonstrate that the neoantigen prognostic risk model is involved in regulating the immune microenvironment, providing a new direction for immunotherapy in ESCC.

We analyzed the immune microenvironment of patients in the high-risk and low-risk groups. The results revealed that the 28 immune cell scores of low-risk patients with better survival rates were significantly lower than those of high-risk patients (Figure 4D). Therefore, we further analyzed the expression of immunogenic cell death (ICD) and immune checkpoint (ICP) markers in different patients. The results showed that the expression levels of ICD and ICP in high-risk patients were higher than in low-risk patients (Figure 4F,G). In ICP, PDCD1 expression was higher in the high-risk group compared with the low-risk group. As a classic immune checkpoint molecule, the high expression of PD-1 indicates that tumor cells may evade immune surveillance by suppressing T cell activity. In ICD, the expression levels of MET and HGF genes were higher in high-risk patients compared with low-risk patients. MET and HGF genes can induce epithelial–mesenchymal transition (EMT) in ESCC, endowing cancer cells with stronger invasiveness and metastatic potential. The above results suggest that the tumor immune microenvironment in high-risk ESCC patients may harbor multiple immune evasion mechanisms, which could promote tumor growth and progression.

### 2.5. Clinical Application of Prognostic Risk Models

**Figure 5:**
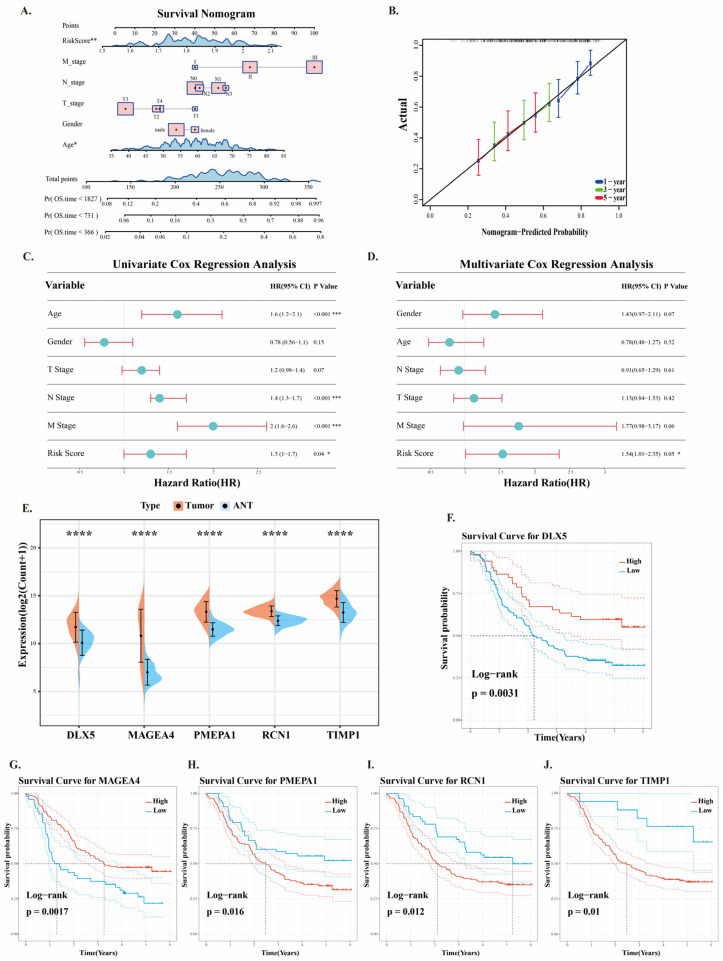
Clinical Performance Evaluation of the ESCC Prognostic Model. (**A**) Nomogram of the prognostic risk model. (**B**) Calibration curves of the nomogram predicting 1-year, 3-year, and 5-year survival rates. (**C**) Univariate Cox analysis of clinical information in ESCC. (**D**) Multivariate Cox analysis of clinical information in ESCC. (**E**) Expression levels of five prognostic genes in the adjacent normal group and cancer groups. (**F**–**J**) KM survival analysis of the five prognostic genes. (* *p* < 0.05; ** *p* < 0.01; *** *p* < 0.001; **** *p* < 0.0001).

We used patients’ different clinical features and risk scores to predict their survival probabilities at 1, 3, and 5 years. The results showed that age and risk score are good predictive factors, providing a relatively accurate assessment of patients’ survival probabilities (Figure 5A). Meanwhile, the 1-year, 3-year, and 5-year survival rate calibration curves demonstrated that the actual survival rates closely matched the survival rates predicted by the nomogram (Figure 5B). It demonstrates that the nomogram has excellent predictive value.

Tumors exhibit heterogeneity, and even the same type of tumor may demonstrate notable biological and clinical characteristic differences among different patients. We performed both univariate and multivariate Cox regression analyses. The univariate Cox analysis results showed that risk score, age, N stage, and M stage were associated with patient prognosis, with the risk score having a correlation coefficient of (*p* = 0.04, HR = 1.3, 95% CI: 1.0–1.7) (Figure 5C). Then, we conducted a multivariate Cox regression analysis and found that the risk score could serve as an independent prognostic factor for ESCC. (*p* = 0.05, HR = 1.54, 95% CI: 1.01–2.35) (Figure 5D). The aforementioned results indicate that the ESCC neoantigen prognostic model has a favorable predictive efficacy.

By integrating the upregulated genes from the GSE53624 dataset with the mutated gene set identified in single-cell RNA sequencing, we successfully constructed a model comprising five key prognostic genes. The analysis of the expression patterns of these genes in cancerous tissues and adjacent normal tissues revealed that they all align with the characteristic upregulation of genes typically observed in ESCC (Figure 5E). To further investigate the impact of these prognostic genes on the survival outcomes of cancer patients, we conducted a Kaplan–Meier survival analysis. The analysis revealed that high expression of DLX5 and MAGEA4 was associated with favorable survival outcomes in patients (Figure 5F,G), whereas elevated expression of the PMEPA1, RCN1, and TIMP1 genes correlated with poor prognosis (Figure 5H–J). This finding is consistent with the expression trends of the prognostic genes in patients with different risk scores (Figure 3H). These results suggest that the higher expression of prognostic genes does not necessarily imply the promotion of tumor progression. On the contrary, high expressions of DLX5 and MAGEA4 appear to be beneficial for patients.

### 2.6. Analysis of Pathway Functions of Five Prognostic Genes in ESCC

**Figure 6:**
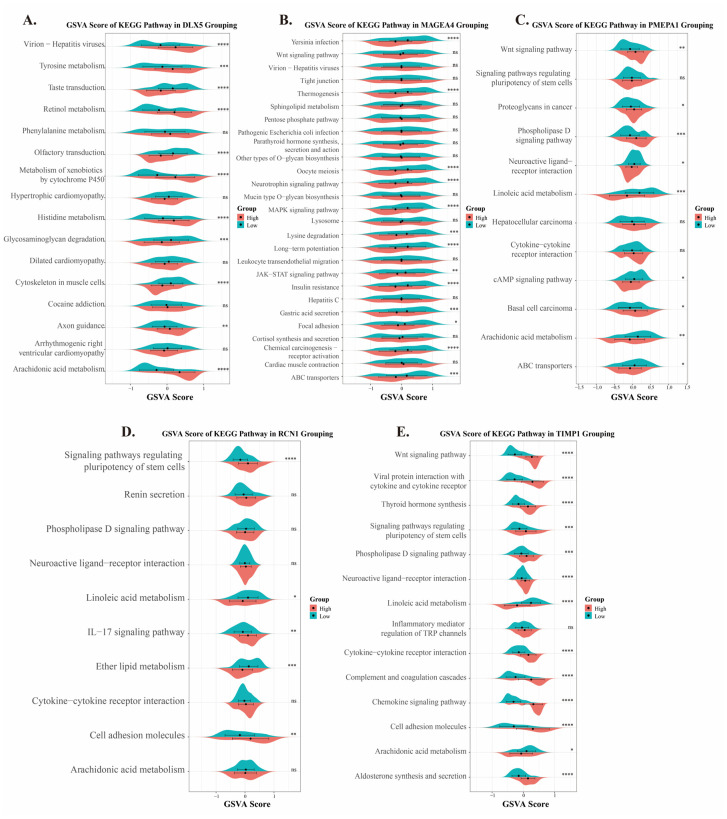
Exploring the regulatory mechanisms of prognostic genes, KEGG pathway enrichment scores of prognostic genes in high-risk and low-risk groups in ESCC. GSVA scores of enriched KEGG pathways for DLX5 (**A**), MAGEA4 (**B**), PMEPA1 (**C**), RCN1 (**D**), and TIMP1 (**E**) in high- and low-risk groups. (* *p* < 0.05; ** *p* < 0.01; *** *p* < 0.001; **** *p* < 0.0001; ns stand for not statistically significant).

To investigate the mechanisms of action of each prognostic gene, we performed KEGG pathway enrichment analysis on the high- and low-risk groups associated with these five prognostic genes, followed by GSVA scoring of the results for each group. The GSVA results for DLX5 showed that the activity of multiple signaling pathways, metabolic pathways, and neural system development pathways was upregulated (Figure 6A and Figure S10A), indicating that DLX5 accelerates the metabolic cycle in the cancer microenvironment by upregulating metabolic pathways, helping cancer cells to better grow and develop. The GSVA results of MAGEA4 indicated that regulatory pathways related to the nervous system, as well as cell signaling and metabolic pathways associated with ESCC, were downregulated (Figure 6B and Figure S10B). MAGEA4 may promote cancer progression by inhibiting the MAPK signaling pathway or through other mechanisms. PMEPA1 upregulates the WNT pathway, metabolic pathways, and others (Figure 6C and Figure S10C), affecting tumor cell proliferation, migration, and survival. It also regulates inflammation responses and the tumor microenvironment, enhancing the invasiveness and treatment resistance of esophageal cancer. Similarly, RCN1 also upregulates the WNT pathway, phospholipase D signaling pathway, and others (Figure 6D and Figure S10D), influencing tumor cell proliferation, migration, and survival while further promoting esophageal cancer invasion and treatment resistance through the regulation of inflammation and tumor microenvironment. Finally, TIMP1 not only inhibits matrix metalloproteinase (MMP) activity, influencing the degradation of the extracellular matrix, but also activates cytokines, immune activation pathways, and the WNT signaling pathway (Figure 6E and Figure S10E), regulating tumor growth, invasion, and immune evasion, playing a crucial role in the development of esophageal cancer. Overall, these five prognostic genes may play a crucial role in the occurrence, progression, and treatment resistance of ESCC by regulating multiple signaling pathways and biological processes.

### 2.7. Prognostic Gene Small Molecule Drug Docking

**Figure 7:**
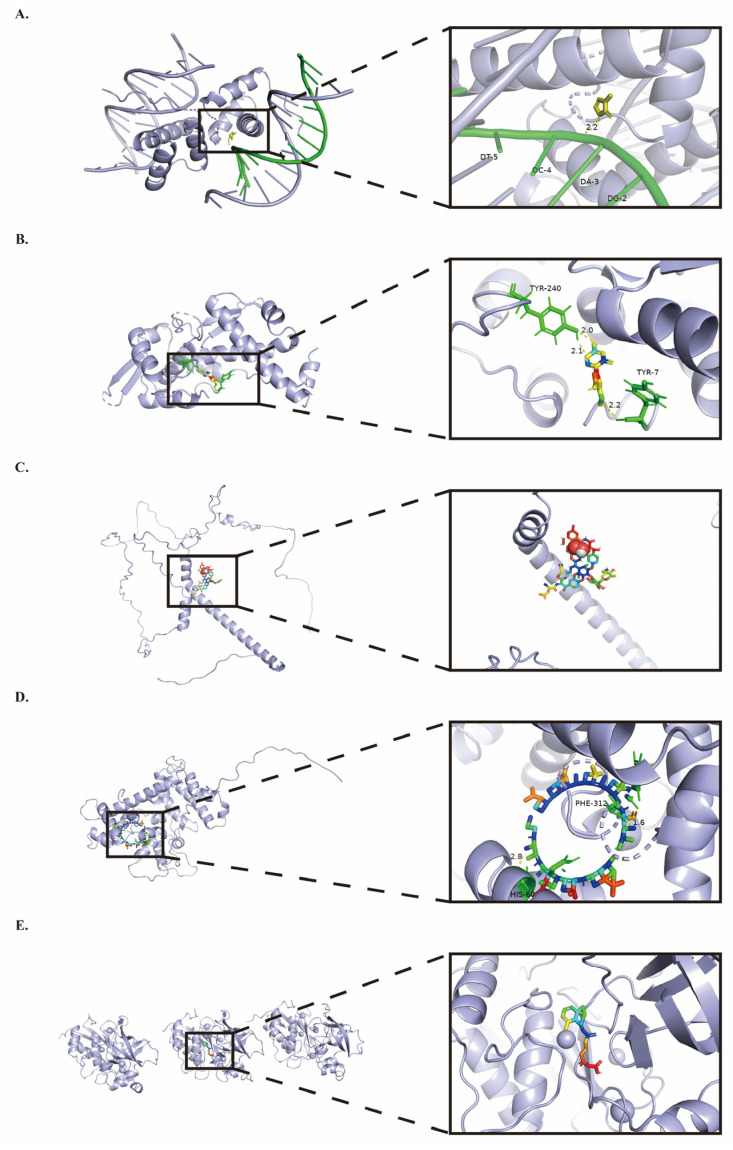
Docking Results of Prognostic Gene-encoded Proteins with Small-molecule Compounds. (**A**) Docking result of DLX5 with Methimazole. (**B**) Docking result of MAGEA4 with Decitabine. (**C**) Docking result of PMEPA1 with Vancomycin. (**D**) Docking result of RCN1 with Cyclosporin A. (**E**) Docking result of TIMP1 with Retinoic Acid.

In this study, we used the CTD database for screening, Autodock molecular docking, and pharmacological studies to identify drugs targeting the prognostic genes. We found that Methimazole tightly binds to DLX5 (Figure 7A) and upregulates DLX5 mRNA expression with a molecular docking simulation binding energy of −4.11 kcal/mol. Methimazole is an anti-thyroid drug primarily used to treat hyperthyroidism, especially in the treatment of autoimmune hyperthyroidism such as Graves’ disease. Decitabine tightly binds to MAGEA4 (Figure 7B) and upregulates MAGEA4 mRNA expression with a molecular docking simulation binding energy of −4.23 kcal/mol. Decitabine is a demethylating agent belonging to nucleoside drugs, primarily used to treat hematological malignancies and inhibit tumor proliferation. It can also restore the expression of normal genes, but bone marrow suppression and infection risks need to be monitored. Vancomycin tightly binds to PMEPA1 (Figure 7C) and downregulates PMEPA1 mRNA expression with a molecular docking simulation binding energy of −15.36 kcal/mol. Vancomycin is an antibiotic primarily used to treat Gram-positive bacterial infections. Its mechanism of action involves inhibiting bacterial cell wall synthesis. Vancomycin is not an anticancer drug and currently does not have any direct indications for cancer treatment. Cyclosporin A can bind tightly to RCN1 (Figure 7D) and downregulate RCN1 mRNA expression with a molecular docking simulation binding energy of −23.79 kcal/mol. Cyclosporin A is a potent immunosuppressant primarily used in organ transplantation and for treating certain autoimmune diseases. It works by suppressing immune responses to prevent organ rejection. Although Cyclosporin A is not an anticancer drug, it has potential in cancer treatment as an adjunct, such as in combination with chemotherapy or in influencing tumor resistance. However, due to its immunosuppressive effects, it may increase the risk of cancer development, so close monitoring is required during clinical use. Retinoic Acid can tightly bind to TIMP1 (Figure 7E) and downregulate TIMP1 mRNA expression with a molecular docking simulation binding energy of −11.54 kcal/mol. Retinoic Acid, a metabolite of vitamin A, is an important endogenous signaling molecule involved in cellular differentiation, proliferation, and apoptosis. It plays a crucial role in medicine, pharmacy, and biology, particularly in the treatment of skin diseases, cancer, and developmental biology. Studies in other cancers (such as breast cancer, liver cancer, and lung cancer) show that Retinoic Acid has the ability to inhibit tumor growth and induce differentiation of tumor cells. It works by regulating cancer-related genes (e.g., p53, RARB) and suppressing tumor cell proliferation. In summary, our findings suggest that the identified five small molecule compounds may have the potential to influence the poor prognosis associated with the five prognostic genes, offering new avenues for further research into adjuvant therapy for ESCC.

## 3. Discussion

Although ESCC can currently be cured through surgical resection combined with postoperative chemotherapy and radiotherapy, patients’ quality of life significantly declines after surgery, and there is still a high recurrence rate and mortality [19]. The complexity and adaptability of the mechanisms involved in the occurrence, progression, and metastasis of ESCC also require the development of additional prognostic and therapeutic approaches. In recent years, immunotherapy has become a groundbreaking strategy in cancer treatment [20]. mRNA vaccines are a powerful form of cancer immunotherapy because they possess high efficiency, specificity, multifunctionality, rapid development, and scalability [15,16]. Neoantigens are the ideal targets for tumor mRNA vaccines [21]; neoantigens are generated by somatic mutations in tumor cells and have specificity that can trigger the body’s antigen-presenting response. This unique property enables researchers to develop more precise targeted therapies [11]. Therefore, screening for mutated genes to identify key neoantigen mutations in ESCC is of significant reference value for patient prognosis and the development of tumor mRNA vaccines [22,23].

In this study, we mined single-cell data to identify mutated genes related to antigen presentation. First, we identified the source of ESCC mutations through manual annotation of single-cell data: the epithelial cell population. This epithelial cell population contains a significant number of malignant epithelial cells [18]. We then separated the CNV cancer cell population from the malignant epithelial cell population using the CopyKAT algorithm. To validate the effectiveness of our cell population separation, we compared the differences between the malignant epithelial cell population and the CNV cancer cell population using classical cancer pathways associated with ESCC. We found that, compared with the remaining malignant epithelial cells, CNV cancer cells are more likely to be regulated by the Wnt and Notch signaling pathways, while the PI3K signaling pathway, which exhibits higher activity in the overall malignant epithelial cells, shows less pronounced regulation of CNV cancer cells compared with other malignant epithelial cells. The WNT signaling pathway plays a crucial role in tumorigenesis, progression, and processes such as cell proliferation, migration, and invasion [24]. The Notch signaling pathway can also promote the epithelial-to-mesenchymal transition (EMT) in ESCC, enabling tumor cells to acquire stronger migration and invasion capabilities [25]. Abnormal activation of the Notch pathway may lead to the proliferation and survival of cancer stem cells, making the tumor more resistant to treatment and prone to recurrence [26]. We found that EPCAM is significantly upregulated in CNV cancer cells, while SFN expression is downregulated. EPCAM is an epithelial cell adhesion molecule involved in intercellular adhesion and signal transduction. It is overexpressed in various epithelial tumors, such as colon cancer, breast cancer, and pancreatic cancer, and its overexpression may enhance the migratory ability of tumor cells [27]. SFN belongs to the 14-3-3 protein family and participates in cell cycle regulation, DNA damage repair, apoptosis, and signal transduction. In ESCC, the downregulation of SFN may lead to cell cycle dysregulation and genomic instability [28]. These results validate the effectiveness of the CopyKAT algorithm in isolating the CNV cancer cell population and reveal the unique regulatory characteristics of CNV cancer cells in signaling pathways, highlighting the complexity of ESCC regulatory mechanisms. These findings provide new directions for further research into the molecular features and functions of CNV cancer cells in ESCC.

We also discovered through pseudotime analysis that the pathway expression varies across different developmental stages of CNV cancer cells. In the early stages of CNV cancer cell development, cancer-related inflammatory responses and developmental pathways are not significantly activated, and immune responses have not yet been triggered. Over time, during the second stage, the antigen presentation signaling pathway, mTOR signaling pathway, and NOD-like receptor signaling pathway in CNV cancer cells were upregulated, promoting tumor angiogenesis, metabolic reprogramming, and metastasis. At the same time, the immune system begins to recognize antigens in the tumor microenvironment and initiates the antigen-presenting response. However, by the final stage, tumors significantly promote their growth, migration, and invasion by upregulating multiple pro-cancer pathways, and the immune system in the body seems unable to suppress this process effectively. This suggests that tumors may inhibit the immune system’s effective response, allowing them to escape immune surveillance and continuously drive their malignant progression. We then further investigated SNP genes in ESCC and found that TP53 biallelic deletion in ESCC is a prerequisite for the development of gene CNAs in the cell cycle, DNA repair, and apoptosis pathways [29]. CNV cancer cells exhibit mutation rates exceeding 65% for both CNV and SNV across different developmental stages, with the mutation rate reaching its peak during the third stage over time. This study revealed the mutational characteristics and pathway changes in CNV cancer cells across different developmental stages, identified high-frequency mutation genes in ESCC, and provided a new method for screening high-frequency mutation genes in cancer.

Neoantigen mRNA vaccine carriers require small molecules of amino acids within the host cells to rely on their own transcription and translation to produce a large number of antigens, thereby effectively triggering the antigen presentation process [11,30]. Therefore, we cross-referenced the upregulated genes and mutated genes in ESCC, resulting in 70 genes. We employed 101 machine learning algorithms to construct the optimal prognostic model screening range. Within this model range, we ultimately selected the LASSO regression, commonly used in medical research, to build the prognostic risk model. The model formula is as follows: Risk Score = 0.0135 × PMEPA1 − 0.0245 × MAGEA4 + 0.0622 × RCN1 − 0.0071 × DLX5 + 0.0792 × TIMP1. PMEPA1 is an androgen-responsive gene initially studied in the context of prostate cancer androgen-regulated gene networks. However, it has since been found to play a role in various types of cancer. The overexpression of PMEPA1 can reduce the effects of linc00941 knockdown. Linc00941, in turn, inhibits the progression of ESCC cells by targeting miR-877-3p [31,32,33]. RCN1 is a calcium-binding protein located in the endoplasmic reticulum lumen, containing six conserved regions. Its main functions include maintaining intracellular homeostasis and regulating cell proliferation and apoptosis. RCN1 plays an important role in the development of various tumors. It is significantly upregulated in ESCC tissues and is closely associated with lymphatic metastasis and poor prognosis. The knockdown of RCN1 significantly inhibits ESCC cell migration, invasion, and epithelial–mesenchymal transition (EMT) and promotes apoptosis of ESCC cells [34,35]. TIMPs (Tissue Inhibitors of Metalloproteinases) are a family of proteins composed of TIMP1, TIMP2, TIMP3, and TIMP4. These proteins are associated with tumor invasion and angiogenesis. The overexpression of TIMP1 or downregulation of TIMP3 is linked to cancer progression and poor prognosis in patients. High levels of TIMP1 in serum are associated with disease progression and poorer prognosis in esophageal cancer (EC) patients [36,37]. MAGEA4 is a cancer–testis antigen primarily expressed in the testes. It interacts with the RING-type ubiquitin ligase RAD18 and activates the DNA synthesis (TLS), which may facilitate tumor evolution. MAGEA4 mediates cancer cell growth by preventing cell cycle arrest and inhibiting p53 transcriptional target-mediated growth suppression [38,39]. DLX5 and DLX6 are two closely related transcription factors. DLX5 is associated with brain development and skeletal development. Additionally, DLX5 has been identified as an upstream regulator of NOTCH1-mediated squamous cell differentiation [40,41]. Mr. Huang and colleagues found that DLX5 is specifically upregulated by SOX2 in ESCC, promoting ESCC cell proliferation and migration abilities [42]. Overall, this model is suitable for various types of diagnostic data and can effectively predict the prognosis and immune therapy response of ESCC patients.

In subsequent research, we validated the correlation between the prognostic risk model and immune infiltration of APCs and MHC II molecules. In the immune microenvironment of ESCC, there is a positive correlation between the prognostic model and immune infiltration of APCs. We found that the MHC molecule scores were higher in high-risk cancer patients compared with low-risk patients. Initially, low-risk ESCC patients do not produce a significant amount of tumor antigens because of their tumor mutations and their immune microenvironment remains relatively inactive. However, as the risk score gradually increases, the immune response in high-risk patients also intensifies, indicating that tumor antigens have been recognized by the immune system. This recognition persists over time. Despite this, the survival rate of high-risk patients remains low. In the high-risk group, ICPs such as CD276, CD86, LAG3, PDCD1, and CTLA4 are highly expressed, inhibiting the activity of CD8+ T cells and CD4+ T cells. As a result, the MHC molecule complex cannot present antigens to CD8+ T cells and CD4+ T cells. Among the 28 immune scores, there is no significant difference in the activity of CD8+ T cells and CD4+ T cells between high-risk and low-risk patients, indicating that CD8+ T cells and CD4+ T cells remain largely inactivated in the cancer immune microenvironment. This process plays a key role in initiating both cellular and humoral immune responses against tumors [43]. In the genes associated with ICD, the high-risk group shows elevated expression of genes such as ANXA1, MET, TLR4, and FPR1. These genes help tumor cells grow and invade while also modulating the body’s immune and inflammatory responses, thereby preventing the tumor from being recognized by the immune system [44]. Based on these results, combining immune checkpoint therapies could improve the survival prognosis and reduce the recurrence probability in high-risk patients.

In terms of personalized treatment for ESCC, we ensured that the ESCC model score could serve as an independent prognostic factor for predicting the outcomes of patients with different clinical characteristics through univariate Cox, multivariate Cox screening, and nomogram analysis. To explore the regulatory mechanisms of prognostic genes, we compared the expression levels of each prognostic gene with survival curves. We found that prognostic genes highly expressed in cancer group patients are not necessarily associated with poorer survival outcomes. The high expression of DLX5 and MAGEA4 genes is associated with better prognostic outcomes, while the overexpression of PMEPA1, RCN1, and TIMP1 predicts poorer prognosis. Our study found that DLX5 mainly upregulates metabolic pathways, accelerating metabolic cycles in the cancer microenvironment and helping cancer cells grow and proliferate more effectively. MAGEA4 primarily encodes tumor-associated antigens, but here, we observed that MAGEA4 affects various neural signal transduction pathways. Downregulating the MAPK signaling pathway and enhancing the signaling of ABO transporters promotes tumor cell proliferation, survival, migration, and immune escape capabilities. PMEPA1 is a gene associated with prostate cancer and other types of cancer, while TIMP1 encodes a metalloproteinase inhibitor. According to pathway enrichment analysis, both PMEPA1 and TIMP1 primarily affect cytokine activity and the classic WNT signaling pathway, playing a crucial role in cancer development. RCN1 mainly functions in the endoplasmic reticulum; however, in ESCC, we found that RCN1 promotes cancer cell proliferation and growth by upregulating squamous epithelial cell development and inflammatory factors within the tumor microenvironment. Understanding the function of each prognostic gene and the mechanisms by which they contribute to poor prognosis is beneficial for developing molecular-targeted treatment strategies.

Targeted drug screening has become a new strategy for disease treatment. With ongoing research into disease mechanisms, various drugs, including valproic acid [45], have been applied in the treatment of cancer. Based on this strategy, we performed targeted drug screening for prognostic genes to provide an alternative treatment option. Methimazole is an anti-thyroid drug commonly used to treat hyperthyroidism (overactive thyroid). It works by inhibiting the synthesis of thyroid hormones, thereby reducing the levels of thyroid hormones in the blood and helping control the symptoms of hyperthyroidism. Methimazole is a thiourea-class drug [46]. Methimazole is not commonly used in cancer treatment, but some studies suggest that certain thiourea-class drugs may influence cancer cells through mechanisms such as affecting cell proliferation and inhibiting the activity of specific enzymes. These effects could potentially contribute to the inhibition of tumor growth or metastasis [47,48]. Decitabine is a chemotherapy drug classified as a DNA methyltransferase inhibitor. It is primarily used to treat certain types of blood cancers, such as acute myeloid leukemia (AML) and myelodysplastic syndromes (MDS). Its mechanism of action involves inhibiting DNA methylation, which leads to the reactivation of silenced genes that are crucial for tumor suppression. By reversing abnormal DNA methylation patterns, Decitabine can inhibit tumor cell growth and promote the expansion of CD8+ effector T cells. This helps enhance the anti-tumor efficacy of therapies, particularly in combination with PD-1 inhibitors, which are used to boost the immune system’s ability to fight cancer [49,50]. Vancomycin is a broad-spectrum antibiotic primarily used to treat serious infections caused by Gram-positive bacteria, especially when other antibiotics are ineffective [51]. While it does not directly treat cancer, cancer patients, especially those undergoing long-term chemotherapy, may experience immunosuppression, which makes them more susceptible to infections caused by resistant bacteria. In such cases, vancomycin can be used to treat bacterial infections in these patients, ensuring that the infection does not interfere with their cancer treatment. Cyclosporin A is an immunosuppressive drug widely used in the treatment of organ transplantation, immune-related diseases, and certain autoimmune disorders. It is a macrolide antibiotic that effectively suppresses the overreaction of the immune system. This helps prevent organ rejection after transplantation and is used to treat immune-related conditions such as rheumatoid arthritis and psoriasis. Cyclosporin A works by inhibiting the activity of T-cells, thus reducing immune responses that could lead to tissue damage or disease progression [52]. Cyclosporin A can tightly bind with RCN1 and downregulate RCN1 mRNA expression. However, Cyclosporin A reduces the body’s immune function, leading to cancer evading immune surveillance. Therefore, there is still considerable research potential for Cyclosporin A in cancer treatment. Retinoic Acid, a metabolite of vitamin A, plays an important role in biological processes such as cell differentiation, proliferation, and apoptosis. Due to its regulatory effects on cell proliferation and differentiation, retinoic acid has been widely studied and applied in cancer treatment, particularly in certain types of blood cancers and skin cancers [53,54]. Retinoic Acid can bind closely with TIMP1, downregulate TIMP1 expression, inhibit tumor growth, and induce tumor cell differentiation. Based on data from the five targeted drugs for the five prognostic genes mentioned above, this approach can help improve the adverse prognosis caused by these genes and enhance patient survival rates.

The innovation of this study lies in the precise identification of CNV cancer cells through the analysis of large-scale single-cell RNA sequencing (scRNA-seq) datasets and the acquisition of CNV characteristic genes using pseudotime analysis. Additionally, we analyzed the SNV gene set of ESCC from the TCGA database and intersected it with the CNV gene set, thereby accurately obtaining the characteristic mutation gene set of ESCC cancer cells. We employed 101 machine learning algorithms to screen the prognostic model range, selected the commonly used LASSO regression within the optimal range to construct the prognostic model, and further optimized the model’s results using elastic net and 100 random seeds. Subsequently, we examined the correlation between the prognostic risk score and immune infiltration of APCs and MHC II molecules to analyze the antigen presentation efficacy of the model. Another innovative aspect of this study is the exploration of the functional roles of each prognostic gene and the investigation of drug-targeting strategies, providing various adjunctive therapeutic options for ESCC. However, there are some limitations in this study. CopyKAT algorithm has certain limitations, and its resolution for CNV detection is constrained by the sparsity of scRNA-seq data and the dynamic range of gene expression, which may result in the inability to detect certain small-scale CNVs. We identified the SNV mutation targets for each prognostic gene, but we did not investigate in depth whether intronic mutations in the TIMP1 prognostic gene could generate novel splicing sites. Currently, we have only identified relevant studies on these five prognostic genes in ESCC through a literature review. Additionally, the limited drug information in the CTD database may have resulted in the omission of higher-quality or more precise targeted drugs. In future research, we plan to integrate more public drug database resources and utilize AI technology to construct a large-scale drug screening model, thereby improving the accuracy and reliability of drug molecular docking.

## 4. Materials and Methods

### 4.1. Data Sources Used for Analysis

In this study, we obtained a scRNA-seq dataset of ESCC from the Gene Expression Omnibus database (GEO, https://www.ncbi.nlm.nih.gov/geo/) (accessed on 11 June 2024) [55], which includes data from 67 patients: GSE145370 (7 patients) and GSE160269 (60 patients). We downloaded bulk RNA-seq data from the Cancer Genome Atlas (TCGA, https://portal.gdc.cancer.gov/) (accessed on 23 June 2024) [56] and the GEO database. The TCGA database includes clinical data for all subtypes of ESCA, and after excluding data from other subtypes, we obtained clinical data for 85 cases. From the GEO database, we obtained 238 clinical data samples from GSE53624 and 120 clinical data samples from GSE53622. scRNA-seq data were merged, and batch effects were removed using Harmony (v1.2.1). Bulk RNA-seq data were merged, and batch effects were corrected using limma (v3.58.1). The ESCC single nucleotide variation (SNV) dataset was downloaded from TCGA. The ESCC-related pathway datasets used for scoring were obtained from the Gene Set Enrichment Analysis database (GSEA, https://www.gsea-msigdb.org/gsea/index.jsp) (accessed on 30 June 2024) [57].

scRNA-seq data and SNV data were primarily used to screen for mutated genes in ESCC cells. Bulk RNA-seq data were used to construct the prognostic model. GSE53624 datasets served as the training set for the prognostic model, while the combined TCGA and GSE53622 datasets were used as the validation set for the prognostic model. The relevant code analysis has been uploaded to the GitHub website at: https://github.com/qqxj/ (accessed on 30 June 2024).

### 4.2. Single-Cell Sequencing Analysis

First, we performed quality control (QC) on the scRNA-seq dataset with the following criteria: (1) Cells with 500 < nFeature_RNA < 5000 were retained; (2) Cells with 500 < nCount_RNA < 35,000 were retained; (3) Cells with percent.mt ≥ 10% were excluded. After quality control, we obtained a final integrated dataset consisting of 703,210 cells. We then performed dimensionality reduction and clustering analysis on scRNA-seq data using Seurat (v4.4.0) across 25 dimensions. The cell subsets were automatically annotated using SingleR (v2.4.1), followed by manual annotation utilizing the CellMarker database [58]. To validate the accuracy of the annotations, we visually displayed the annotated marker genes using a bubble plot.

### 4.3. The Aneuploid Cell Population in ESCC

The original GSE160269 dataset contains 44,730 malignant epithelial cells, which is close to the number of epithelial cells we annotated [18]. We used CopyKAT (v1.1.0) to infer copy number variations (CNVs) in epithelial single cells, distinguishing aneuploid cells from euploid cells. Aneuploid cells are generally considered part of tumor cell mutations, and we defined them as CNV cancer cells. Subsequently, we used AUCell (v1.24.0) to evaluate the differences in the activity of CNV cancer cells and malignant epithelial cells in classical cancer-related pathways of ESCC.

### 4.4. Pseudotime and Somatic Mutation Analysis

To analyze the mutations in CNV cancer cells along the developmental trajectory, we used Monocle (v2.30.1) to construct the growth and developmental trajectory of tumor cells. This allowed us to divide the tumor cells into three developmental trajectories representing different time points. This algorithm is based on machine learning techniques and uses a set of specific genes as input to arrange cells into a trajectory structure with branching points. The analysis results show that different stages of development represent CNV cancer cell trajectories at different time points of growth. Building on this, we further used GSEA to evaluate the enrichment of cells in immune-related pathways under different states. Using maftools (v2.18.0), we analyzed the SNV dataset of ESCC from the TCGA database to identify SNV genes. We then intersected the SNV gene set obtained from the TCGA database with the CNV feature gene set derived from single-cell data, resulting in the identification of mutation genes specific to ESCC. Furthermore, we calculated the mutation rate of CNV cancer cells at each stage: the number of mutated genes in CNV cancer cells at each developmental stage divided by the number of genes that have both CNV and SNV at that stage, multiplied by 100%, equals the mutation rate of genes in CNV cancer cells that have both CNV and SNV at each developmental stage. Based on this analysis, we selected epithelial tumor cells that exhibited both SNV and CNV to define a gene set of high-frequency mutations in ESCC.

### 4.5. Constructing a Neoantigen Prognostic Risk Model

We conducted a differential analysis between the cancer group and the adjacent normal group in the GSE53624 dataset, defining the upregulated genes in the cancer group as overexpressed genes in ESCC. The intersection of the overexpressed gene set from the GSE53624 dataset and the characteristic mutation gene set of ESCC was used as the training set for the ESCC neoantigen prognostic risk model. The combined TCGA dataset and the GSE53622 dataset were utilized as the validation set for this model. First, we used 10 machine learning algorithms: supervised principal component analysis (SuperPC), generalized boosted regression model (GBM), survival support vector machine (Survival-SVM), elastic network (Enet), Cox partial least squares regression (plsRcox), least absolute shrinkage and selection operator (LASSO), ridge regression, stepwise Cox regression, Cox boost, and random survival forest (RSF), and constructed 101 machine learning algorithm models. The consistency index (C-index) and average C-index of the training and validation sets of each model were analyzed [59,60]. By comparing the ideal model with the average C-index model, we selected the LASSO regression method, commonly used in medical research for constructing prognostic models, from the best-performing model range. To optimize the model’s results and accuracy, we used Elastic Net and 100 random number seeds. To optimize the sample sizes of the training and validation sets, we employed the bootstrap random sampling method, expanding the samples in both sets to 500 [61,62]. The size of the penalty term is controlled by two parameters, λ and ρ. Here, we used caret (6.0.94) and glmnet (4.1.8) to select the optimal ρ and λ for identifying stable and reliable neoantigen genes in ESCC, which were then used to construct the neoantigen risk model. Finally, we used the receiver operating characteristic (ROC) curve to validate the model’s performance. We evaluated the independent predictive ability of the prognostic risk model through nomogram, univariate Cox, and multivariate Cox analyses.

### 4.6. Antigen Presentation Effect

We used the Tumor Immune Estimation Resource (TIMER, https://cistrome.shinyapps.io/timer/) (accessed on 22 June 2024) to analyze the correlation between prognostic-related genes and APCs [63]. We utilized ggcorrplot (v0.1.4.1) to investigate the correlation between the prognostic risk model and immune infiltration of APCs and MHC II-related genes.

### 4.7. Immune Microenvironment

To assess the relationship between the prognostic risk model and immune infiltration, we used the single-sample Gene Set Enrichment Analysis (ssGSEA) algorithm from GSVA (v1.50.5) to calculate the immune infiltration levels of 28 immune cell types. This allowed us to observe the relationship between the prognostic risk model and immune infiltration. We also examined the expression levels of immune checkpoint inhibitors and immunogenic cell death-related genes in both the cancer group and the adjacent normal group.

### 4.8. Pathway Analysis of Prognostic Genes in High- and Low-Risk Groups

To further investigate the adverse prognostic factors associated with the prognostic genes, we categorized the top 30% and bottom 30% of patients based on the expression levels of the prognostic genes in the cancer group of the training set as the high-risk group and the low-risk group, respectively. We then analyzed the differences between the groups and evaluated changes in pathway activity using Gene Set Variation Analysis (GSVA).

### 4.9. Small Molecule Drug Screening and Docking

To regulate the expression of prognostic genes, the drug selection criteria focus on the survival curves of cancer patients and the expression levels of prognostic genes. First, we downloaded a small molecule library interacting with the prognostic genes from the Comparative Toxicogenomics Database (CTD) [64] and retrieved small molecule structures from the PubChem database [65]. Next, we searched and downloaded the biomolecular structures translated by the prognostic genes from the UniProt database [27]. We used Autodock (Linux, v4.2) for molecular docking to explore the interaction between small molecules and the prognostic genes. Finally, based on the criteria, we performed automatic docking of the biomolecules and small molecule compounds. The significant interactions between small molecules and biomolecules during the docking process were determined by the lowest binding energy. The docking results were visualized using PyMol (v2.8, open-source).

### 4.10. Statistical Methods

We used R (v4.3.2) for statistical analysis. For intergroup differences, non-parametric tests were employed, and the significance of survival probabilities between samples was assessed using the log-rank test. Statistical significance was determined based on *p* < 0.05 or FDR < 0.05. Correlation analysis was performed using Spearman’s rank correlation method.

## 5. Conclusions

In this study, we integrated scRNA-seq data with SNV mutation data to identify high-frequency mutation genes in CNV epithelial cancer cells at different developmental stages in ESCC. We Grammatical errors have now been intersected the high-frequency mutation dataset with the upregulated genes in ESCC and utilized 101 machine-learning algorithms to construct a neoantigen prognostic risk model. This model effectively predicts patient prognosis and immune efficacy, and its antigen presentation capability was further validated by examining the correlation between risk scores and immune infiltration of APCs and MHC II molecules. Additionally, we explored the mechanisms linking prognostic genes to poor outcomes, revealing how they contribute to tumor malignancy by regulating processes such as cancer cell proliferation, migration, and immune evasion. We further identified potential drugs associated with these prognostic genes, providing new insights for targeted therapies. This study offers theoretical support for the development of mRNA vaccines, personalized treatments, and novel targeted drugs for ESCC patients.

## Data Availability

All raw data in this article are from the GEO database and the TCGA database, and the contact information is sunyj6039@163.com.

## Supplementary Materials

The following supporting information can be downloaded at: https://www.mdpi.com/article/10.3390/ijms26073373/s1.

## Abbreviations

The following abbreviations are used in this manuscript:

ESCC	Esophageal Squamous Cell Carcinoma
EAC	Esophageal Adenocarcinoma
APC	Antigen-presenting cells
DC	Dendritic cells
CTL	Cytotoxic T lymphocyte
SNV	Single Nucleotide Variant
CNV	Copy Number Variation
GSEA	Gene Set Enrichment Analysis
SuperPC	Supervised Principal Components
GBM	Gradient Boosting Machine
Survival-SVM	Survival Support Vector Machine
Enet	Elastic Net
PlsRcox	Partial Least Squares Regression Cox
LASSO	Least Absolute Shrinkage and Selection Operator
RSF	Random Survival Forest
ROC	Receiver Operating Characteristic
TME	Tumor Microenvironment
GSVA	Gene Set Variation Analysis
ssGSEA	Single-sample Gene Set Enrichment Analysis
ICI	Immune Checkpoint Inhibition
ICP	Immune Checkpoint Proteins
OS	Overall Survival
MET	Mesenchymal-Epithelial Transition
LD	Linear dichroism
ESCA	Esophageal carcinoma
