# Supplementary material for: 101 Machine Learning Algorithms for Mining Esophageal Squamous Cell Carcinoma Neoantigen Prognostic Models in Single-Cell Data
Source: Int J Mol Sci. 2025 Apr 4;26(7):3373. doi: 10.3390/ijms26073373 (PMC11989522; doi:10.3390/ijms26073373)
Supplement: Supplementary file 1 [file ijms-26-03373-s001.zip › Supplementary figure legend.pdf]

Supplementary figure legend

Figure S1. Experimental Flow Chart (A) Overall Process Analysis (B) Process of Screening Neoantigen Genes

Figure S2. Pathway Activity Scores in Epithelial Cell Clusters. (A) Wnt Signaling Pathway Score (B) NOTCH Signaling Pathway Score (C) PI3K-AKT-mTOR Signaling Pathway Score

Figure S3. Removal of batch effects in bulkRNA-seq data. (A) PCA plot before merging TCGA and GEO data (B) PCA plot after merging TCGA and GEO data

Figure S4. Validation results of the new neoantigen prognostic model. (A) KM curve of high and low-risk groups in the validation set (B) ROC curve in the validation set （C） Scatter plot of patient survival distribution in high and low-risk groups, and expression distribution of five prognostic genes

Figure S5. Display of results from stepwise backward Cox regression and random forest algorithm. (A) Stepwise backward Cox regression selection results (B) Random forest selection results （C） KM curve of high and low-risk groups in the validation set (D) ROC curve in the validation set

Figure S6. Immune Predictive Performance of the ESCC Prognostic Model. (A) Immune Infiltration Scores of MHC II Molecules in High- and Low-Risk Patient Groups (B) DLX5 (C) MAGEA4 (D) PPMEA1 (E) RCN1 (F) Correlation Analysis with APCs (*P < 0.05; **P < 0.01; ***P < 0.001; ****P < 0.0001).

Figure S7. Exploring the regulatory mechanisms of prognostic genes, GO pathway enrichment scores of prognostic genes in high-risk and low-risk groups in ESCC.GSVA scores of enriched KEGG pathways for DLX5 (A), MAGEA4 (B), PMEPA1 (C), RCN1 (D), and TIMP1 (E) in high- and low-risk groups. (*P < 0.05; **P < 0.01; ***P < 0.001; ****P < 0.0001).

Figure S8. Heatmap representation of diploid and aneuploid cells distinguished by CopyKAT analysis.

Figure S9. Expression of marker genes in squamous epithelial carcinoma cells. (A) Bubble plot showing the expression of EPCAM and SFN in the two cell populations. (B) Differential expression of EPCAM and SFN between the two cell groups.

Figure S10.Sensitivity analysis of different Copykat algorithm thresholds (ngene.chr=5 is the default true value)
