# Supplementary figures and images for: 101 Machine Learning Algorithms for Mining Esophageal Squamous Cell Carcinoma Neoantigen Prognostic Models in Single-Cell Data
Source: Int J Mol Sci. 2025 Apr 4;26(7):3373. doi: 10.3390/ijms26073373 (PMC11989522; doi:10.3390/ijms26073373)

A.

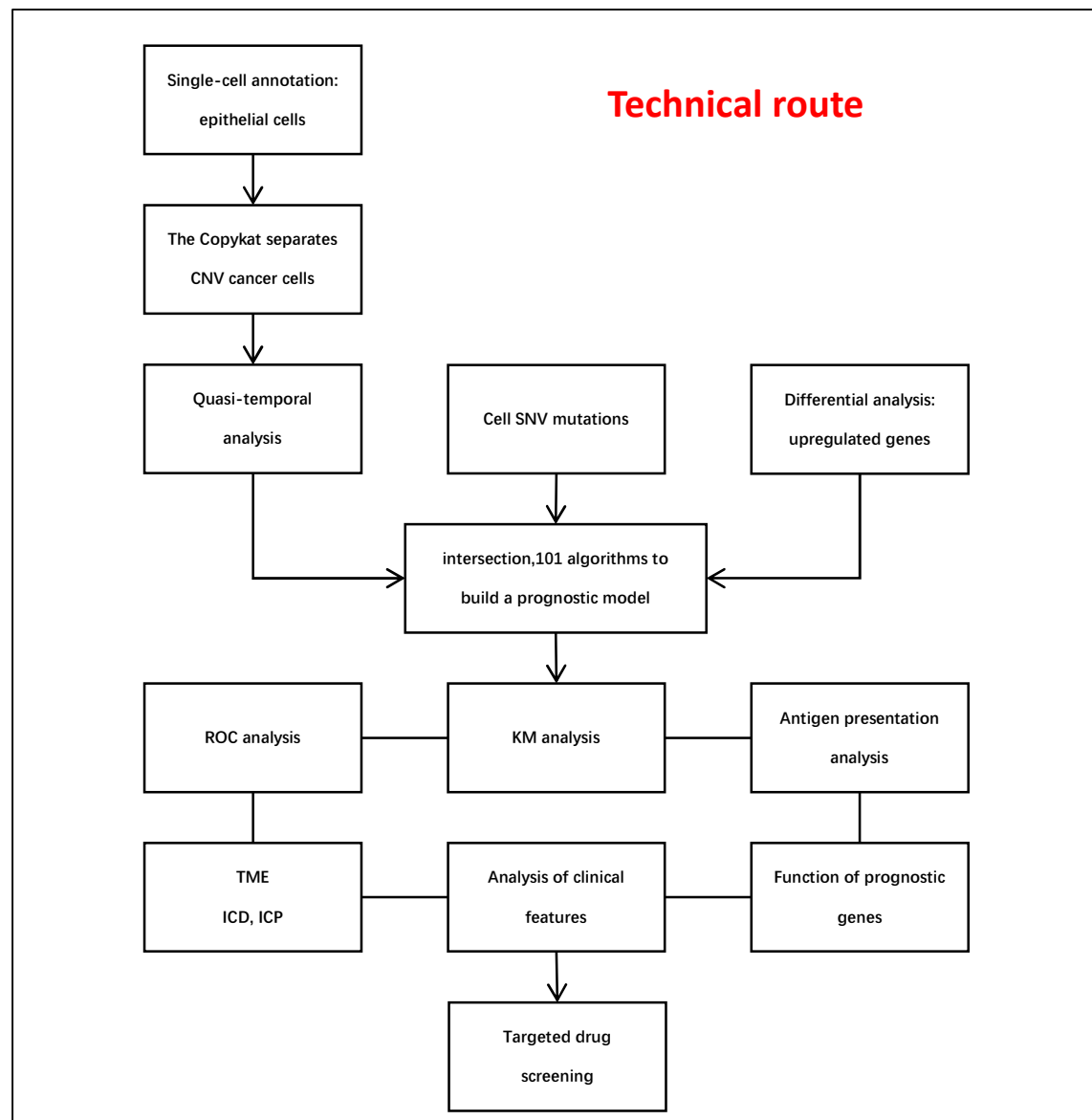

B.

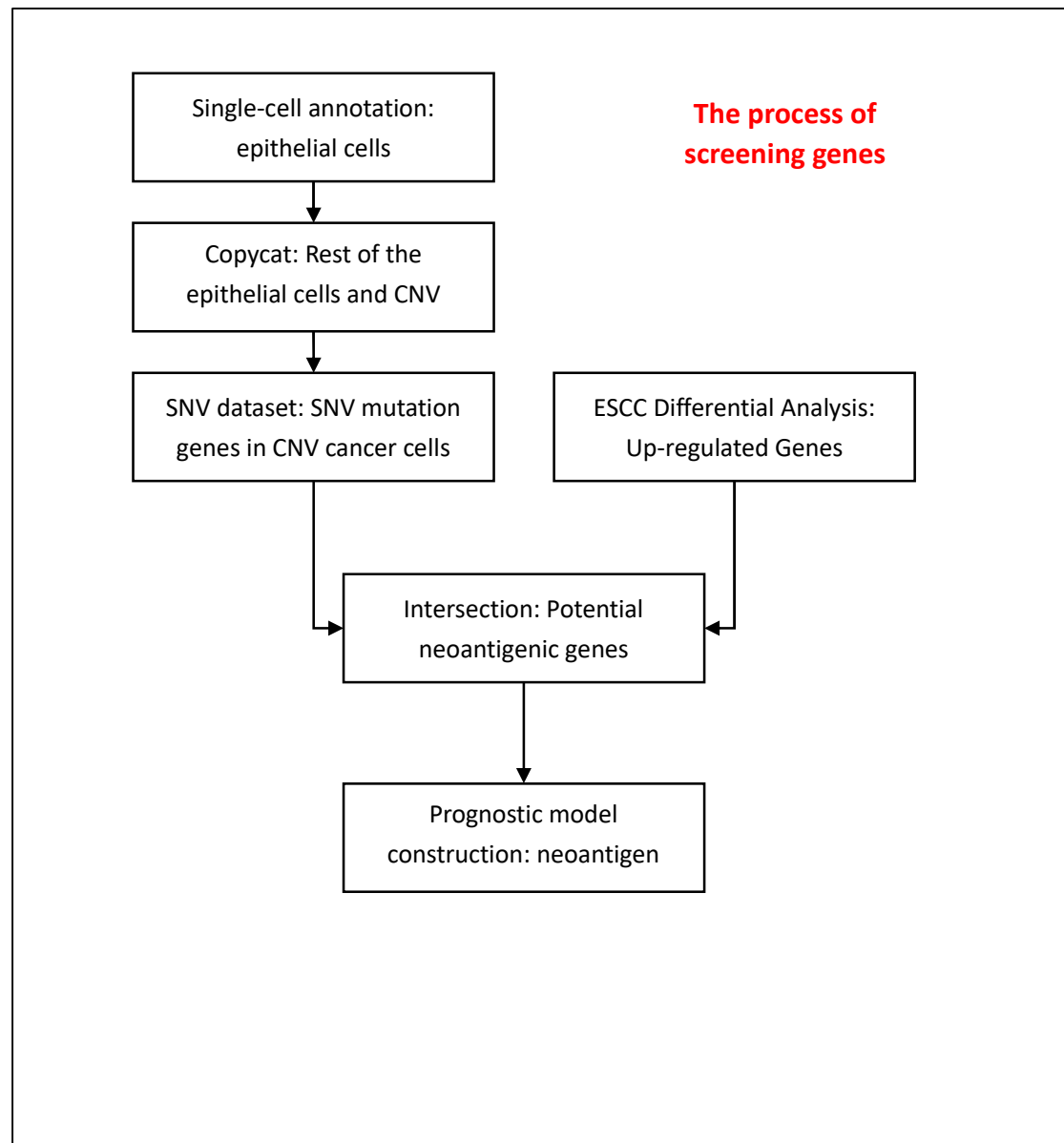

Supplement: Supplementary file 1 [file ijms-26-03373-s001.zip › s1.pdf]

Sensitivity of Different Thresholds

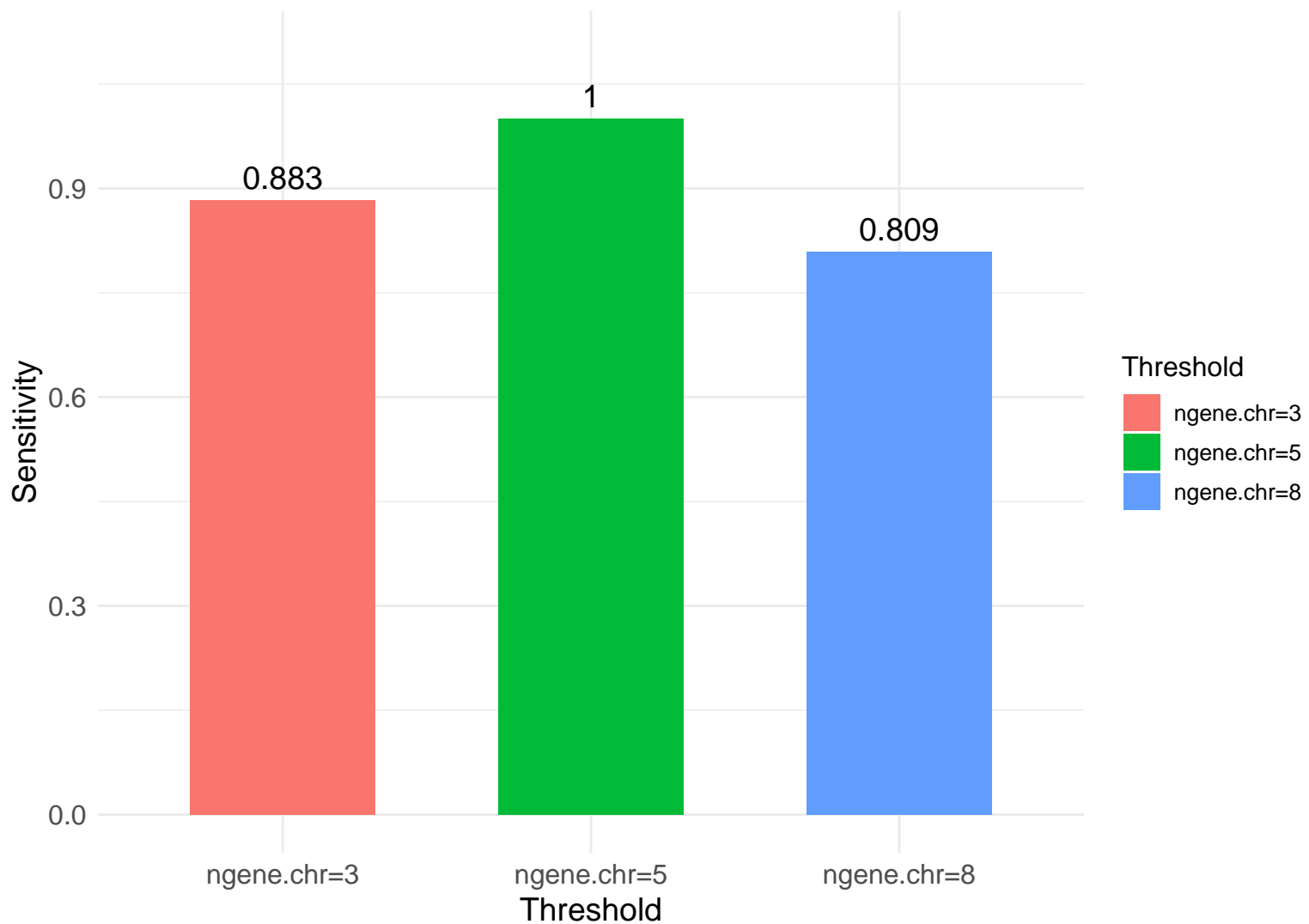

Supplement: Supplementary file 1 [file ijms-26-03373-s001.zip › s10.pdf]

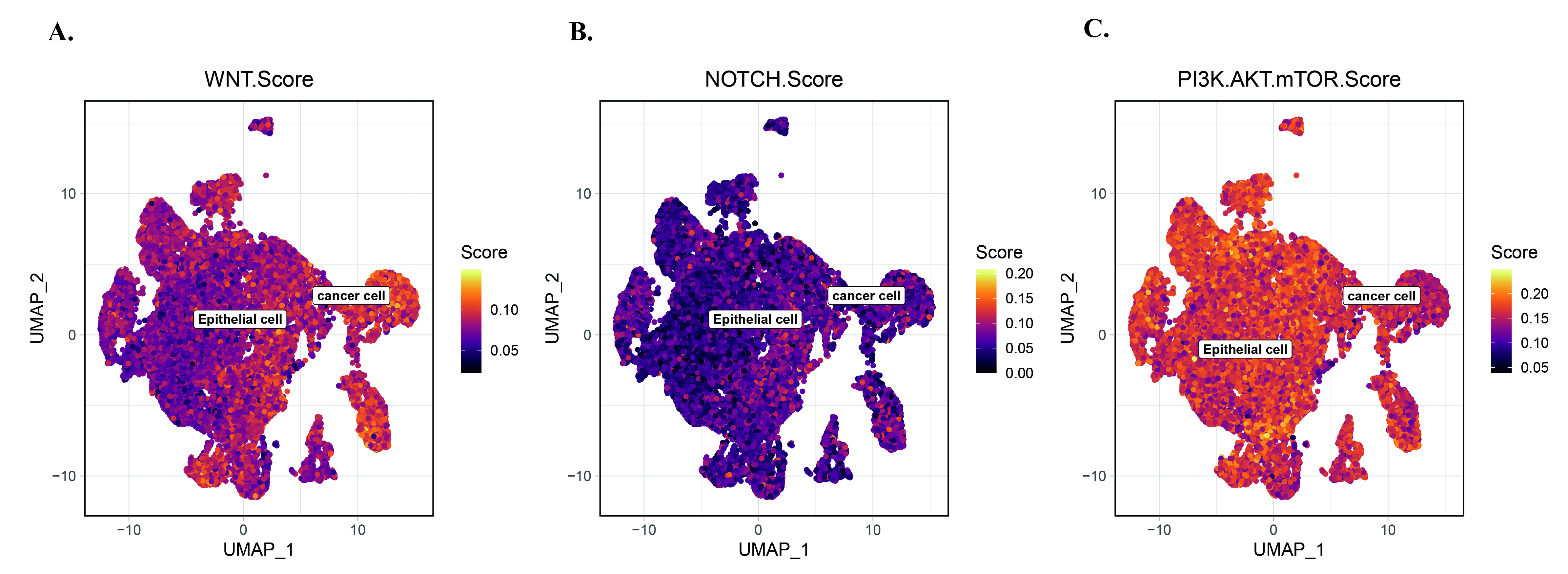

Supplement: Supplementary file 1 [file ijms-26-03373-s001.zip › s2.tif]

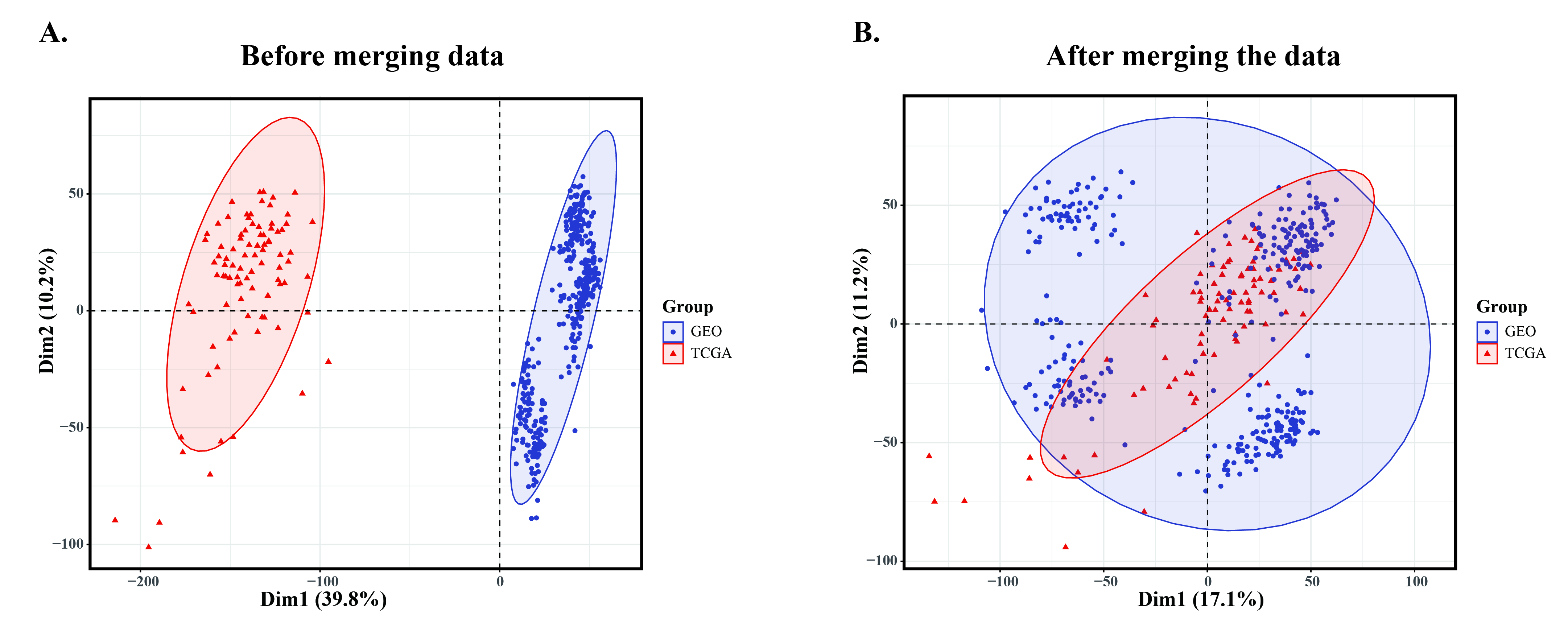

Supplement: Supplementary file 1 [file ijms-26-03373-s001.zip › s3.tif]

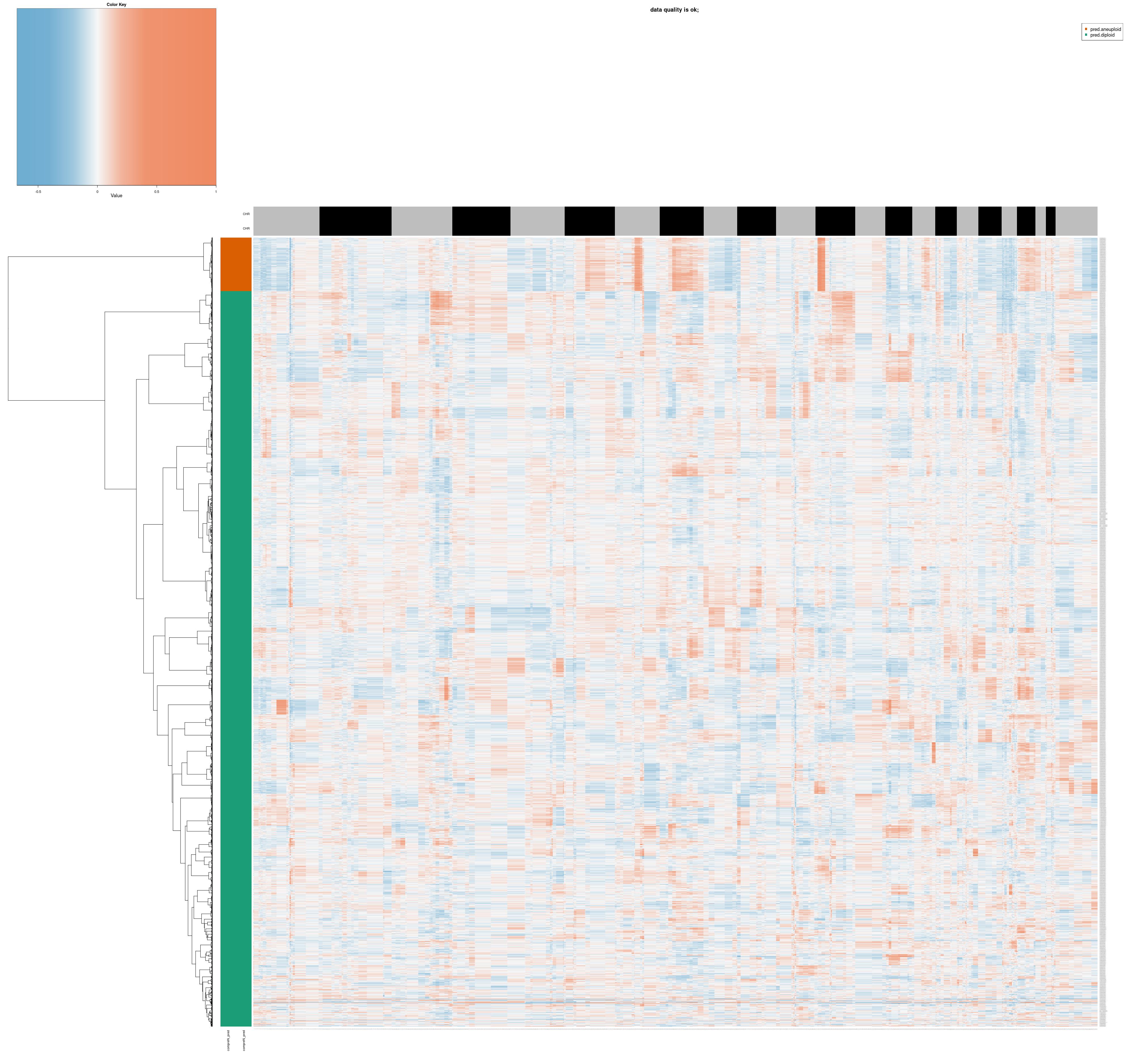

Supplement: Supplementary file 1 [file ijms-26-03373-s001.zip › s8.jpeg]
